# Is implementation research out of step with implementation practice? Pathways to effective implementation support over the last decade

**DOI:** 10.1177/26334895221105585

**Published:** 2022-06-06

**Authors:** Allison Metz, Todd Jensen, Amanda Farley, Annette Boaz

**Affiliations:** 1 School of Social Work, 2331 University of North Carolina at Chapel Hill, Chapel Hill, NC, USA; 2 Department of Health Services Research and Policy, 4906 London School of Hygiene and Tropical Medicine, London, UK

**Keywords:** implementation practice, evidence-based, implementation supports, implementation

## Abstract

**Background:** There is growing interest in the lived experience of professionals who provide implementation support (i.e., implementation support practitioners). However, there remains limited knowledge about their experiences and how those experiences can contribute to the knowledge base on what constitutes successful and sustainable implementation support models. This study aimed to examine pathways of implementation support practice, as described by experienced professionals actively supporting systems’ uptake and sustainment of evidence to benefit children and families. **Methods:** Seventeen individuals with extensive experience providing implementation support in various settings participated in semi-structured interviews. Data were analyzed using qualitative content analysis and episode profile analysis approaches. Iterative diagramming was used to visualize the various pathways of implementation support practitioners’ role reflection and transformation evidenced by the interview data. **Results:** Findings highlighted rich pathways of implementation support practitioners’ role reflection and transformation. Participants described their roots in providing implementation support as it relates to implementing and expanding the use of evidence-based programs and practices in child and family services. Almost all participants reflected on the early stages of their careers providing implementation support and described a trajectory starting with the use of “push models,” which evolved into “pull models” and eventually “co-creation or exchange models” of implementation support involving both technical and relational skills. **Conclusions:** Developing an implementation support workforce will require a deeper understanding of this lived experience to prevent repeated use of strategies observed to be unsuccessful by those most proximal to the work. The pathways for implementation practice in this study highlight impressive leaps forward in the field of implementation over the last 15 years and speaks to the importance of the professionals leading change efforts in this growth.

**Plain Language Summary:**

Over the past few years, professionals in the field of implementation science have identified a growing gap between implementation research and implementation practice. While this issue has been highlighted informally, the field is lacking a shared understanding and clear way forward to reconcile this gap. In this paper, the authors describe how professionals providing implementation support have shifted their implementation practice over time through systematic observations of what works (and what does not work) for supporting and sustaining evidence use in service systems to improve population outcomes. The authors share the impressive leaps forward made in the field of implementation practice – from didactic training to responsive and tailored implementation strategies to co-created and relationship-based implementation solutions. The paper concludes with a call to action to the field for the creation of a virtuous learning cycle between professionals conducting implementation research and professionals providing implementation support to change practice as a way to produce a more robust and relevant science of implementation.

## Background

The field of implementation science has highlighted the importance of active implementation through the use of specific implementation frameworks and models (Greenhalgh et al., 2004; Metz et al., 2015; Metz & Bartley, 2012; Rycroft-Malone, 2004, 2013) to support and sustain use of research evidence (URE) in human services. Specific approaches and models for providing implementation support have also been identified, including facilitation, brokering, and consultation (Beidas et al., 2012; Hering, 2016; Kirchner et al., 2016; Ritchie et al., 2020). Implementation support practitioners (ISPs) carry out these approaches by serving in the role of facilitators, coaches, knowledge brokers, and technical assistance providers to support implementation of evidence-informed programs and practices and to sustain and scale research evidence for improved and equitable population outcomes (Albers et al., 2020). There has been increased interest in the knowledge, skills, and attitudes needed by these professionals to effectively provide implementation support (Metz et al., 2021; Bührmann et al., 2022). Moreover, identifying competencies for ISPs has been described as critical for growing a workforce that can help to close the research-to-practice gap in service to improved population outcomes (Straus et al., 2011).

Recent literature in the field of implementation science demonstrates a particular growing interest in the lived experience of ISPs, including how they learn and adapt their implementation support skills and select implementation strategies based on their experiences, and how they remain resilient in the face of adaptive and ongoing implementation challenges in the complex systems in which they work. For example, Olmos-Ochoa et al. (2021a) describe how reflective writing can support continuous learning among ISPs, specifically supporting professionals who facilitate implementation, by enabling them to evaluate their facilitation process, identify areas for improvement, and support their learning and effectiveness. In a different study, the emotional labor and affective experiences of ISPs are documented (Olmos-Ochoa et al., 2021b) and related to facilitator effectiveness, implementation outcomes, and ultimately the workforce of professionals who provide implementation support. These studies point to the relational processes and affective experiences of providing implementation support. As ISPs attend to both the technical and relational needs of implementing staff and leadership, they expend emotional, physical, and mental energy. The field of implementation is more routinely acknowledging the wide range of skills and attributes, both technical and relational, needed to effectively, and resiliently, provide implementation support (Albers et al., 2020; Black et al., 2021; Leeman et al., 2017; Moore et al., 2018).

However, there remains limited knowledge about the experiences of ISPs and how their experiences can contribute to the knowledge base on what constitutes both successful and sustainable implementation support models. The URE field has documented trajectories in implementation support from early “push models” that attempted to move research evidence into communities through didactic training without a focus on stakeholders or context to “pull models” that endeavor to respond to community needs through appropriately matched implementation support strategies, with a more recent emphasis on “co-creation or exchange models” that create reciprocal dialogue among implementers, stakeholders, researchers, and ISPs to improve the use of contextualized and relevant evidence in practice (Metz et al., 2019). Exchange models in complex systems require learning among different stakeholders on how elements of the system are inter-related to produce current outcomes (Senge, 2014). Few studies, though, have addressed how ISPs have changed their approaches to supporting implementation of evidence-informed programs and practices over the course of their careers, why they made these changes in their approach to implementation support, and what we can learn from this as we seek to grow a workforce that can provide effective implementation support in complicated and complex service systems.

The purpose of this study was to examine pathways of implementation support practice, as described by experienced professionals actively supporting service systems to uptake and sustain evidence to improve outcomes for children, youth, and families. Through interviews, ISPs reflected on their experiences providing implementation support and described factors that influenced changes in their approaches overtime to increase the effectiveness of their implementation support.

Understanding what it takes to provide implementation support from the perspective of those hired or contracted to take on this role will help us close the gap between implementation science knowledge and its application in human services. Indeed, the perceived gap between implementation research and implementation practice has become a growing conversation in the field (Ritchie et al., 2020; Rapport et al., 2021). Grounding ourselves in the lived experiences of successful and long-term ISPs can help to improve the relevance of implementation research and its accompanying theory, models, frameworks, and strategies for professionals seeking to create change and support evidence use in complex systems. Understanding the perspectives and experiences of ISPs will also help us identify strategies that limit professional burnout for those providing implementation support in complex systems where everyday challenges such as staff turnover, shifting policy priorities, and changes in funding can inhibit their ability to successfully support change and evidence use (Metz et al., 2021).

## Methods

### Study Setting and Participants

The current study prioritized the perceptions and experiences of individuals in the United States with relatively extensive professional experience (i.e., 15 + years) providing implementation support in public child welfare, early childhood, and children's mental health to support the URE. Consistent with definitions articulated by Koerber and McMichael (2008) and parameters of purposive sampling highlighted by Curtis et al. (2000), we employed a hybrid purposive-convenience sampling approach to recruit and engage participants. Our approach was purposive in the sense that we strategically sought out “participants who possess certain traits or qualities” (Koerber & McMichael, 2008, p. 464). Moreover, we prioritized the following parameters of purposive sampling: (a) the potential to generate rich information, (b) the potential to generate believable explanations, and (c) feasibility in terms of time, resources, and the ability of the research team to relate to participants and their experiences (Curtis et al., 2000).

An ability to relate to participants seemed paramount given the general complexity and nuance of the work in which ISPs engage (Albers et al., 2020). Consequently, our approach was convenient in the sense that we capitalized on our professional network with individuals who had extensive experience providing implementation support, particularly in child welfare contexts. Although aspects of convenience sampling can be categorized as a limitation—largely due to implications for the analytic generalizability of findings—we echo the sentiments of Koerber and McMichael (2008) that “the same close relationship between researcher and research site that makes a sample convenient often grants the researcher a level of access to and familiarity with the sample that guarantees a richness of data that could not be attained if the sample were less familiar, and therefore less convenient, to the researcher” (p. 463).

Our final analytic sample consisted of 17 individuals, each with extensive experience providing implementation support in various settings, including public child welfare, early childhood, and children's mental health (see Table 1 for more details on participant characteristics). To gauge the optimal number of participants, we attended to general data saturation, or the extent to which new interviews demonstrably failed to produce substantively novel themes or concepts (Guest et al., 2006).

**Table 1.** Participant characteristics.

	*n*	%
**Gender identity**		
Female	14	82%
Male	3	18%
**Racial/Ethnic identity**		
Non-Hispanic White	14	82%
African American/African Descent	2	12%
Hispanic White and Asian	1	6%
**Years of professional experience**		
15 +	14	82%
6–10	2	12%
11–15	1	6%
**Focus of work (check all that apply)**		
Child welfare	12	71%
Mental and behavioral health	9	53%
Implementation science	8	47%
Criminal justice	5	29%
Public health	4	24%
Health	3	18%
Other	3	18%
K-12 education	2	12%
**Work setting (check all that apply)**		
Non-profit	6	35%
Higher education	5	29%
Local government	5	29%
State government	4	24%
Other	4	24%
Federal government	1	6%
For-profit	1	6%

Almost all participants (*n* = 16) provided implementation support from outside of the direct service delivery system, being based either within university implementation centers (*n* = 7) or in nonprofit, intermediary organizations (*n* = 9). The remaining participant who provided implementation support from within the service delivery system was selected for this role based on previous experience working at a university-based implementation center. All participants had long-term experience using implementation science frameworks to support change efforts. The implementation support role has been described as part of the “service delivery support system” (Wandersman et al., 2008) and has been expanded upon in recent years to include the roles of implementation facilitators (Kirchner et al., 2014; Kirchner, et al., 2016; Parker, et al., 2014) and ISPs (Albers et al., 2020; Albers et al., 2021; Metz et al., 2021).

### Data Collection Procedures

Data were collected via in-depth, semi-structured interviews. Interviews were 60 min in duration, on average. The Zoom platform was used to engage with participants and record interviews. Interview prompts were developed in alignment with the following core foci: (a) implementation support strategies used to support the URE, (b) the extent to which implementation support strategies involve stakeholders and for what purpose, and (c) under what conditions specific implementation support strategies, including strategies that foster stakeholder engagement, contribute to supporting the use of research evidence. Participants were also encouraged to reflect on how their approach to providing implementation support has changed over the course of their careers—a topic that represents a core focus of the current study.

Participants were sent the interview guide prior to their interview in the event they desired to familiarize themselves with the interview prompts. One member of the research team (i.e., first author) led the interviews, and two other members of the research team (i.e., second and third authors) attended the interviews to observe and engage in general notetaking. Participants had the opportunity to provide their informed consent verbally; the Institutional Review Board at the authors’ University reviewed all study protocols and designated the project with exempt status. Audio recordings from each interview were transcribed verbatim, in preparation for analysis.

### Data Analysis

The larger frame of our analysis was informed by the *sort and sift, think and shift* strategy as articulated by Maietta et al. (2021), which represents an iterative process whereby data analysts dive into their data to “understand its content, dimensions, and properties, and then step back to assess what they have learned and to determine next steps” (p. 2045). To begin, our initial analysis followed a *qualitative content analysis* approach as outlined by Schreier (2014). This framework emphasizes the development and use of a coding frame whereby one or more main categories are specified (i.e., structuring); main categories “are those aspects of the material about which the researcher would like more information” (Schreier, 2014, p. 174). This structuring is followed by the identification of multiple subcategories (i.e., generating), which “specify what is said in the material with respect to these main categories” (Schreier, 2014, p. 174). The first, second, and third authors engaged in coding activities using Dedoose software.

Our main conceptual categories centered on (a) implementation support strategies used to support evidence use, (b) stakeholder engagement in the context of providing implementation support, and (c) conditions that support the provision of implementation support and evidence use. Consistent with a qualitative analytic approach, our analysis began immediately and organically during the interviewing process (Creswell & Poth, 2018). The research team met together following each interview to discuss insights and observations, summaries of which were placed into a project memo document for later reflection and use (Birks et al., 2008).

Following the initial phase of data analysis, the research team reflected on emergent findings and saw value in examining participants’ perceptions about change or continuity in their approaches to implementation support over time—what we came to refer to as “ISP role reflection and transformation.” To accommodate this vantage point, we employed narrative analysis and the development of episode profiles (Maietta et al., 2021; Oliver, 1998), which enabled us to code and analyze the full interview text for each participant as the focal unit of analysis. Diagramming was used to begin visualizing the various pathways of ISP role reflection and transformation evidenced by the interview data (Hamilton et al., 2011). Iterative versions of the diagram served as an object for discussion among members of the research team, allowing us to evaluate (a) the extent to which interview data corroborated each element in the diagram and (b) whether interview data suggested the inclusion of additional diagram elements. A final diagram resulted from consensus being reached among all members of the research team.

## Results

Almost all participants reflected on providing implementation support for evidence-based program and practices in the early stages of their careers and described the use of “push models” that relied on unidirectional support such as didactic training of practitioners without a focus on stakeholders or context. The majority of participants described an evolution to “pull models” where their implementation support was more focused on the needs of end users and emphasized contextual fit analysis involving participatory approaches, as well as multi-level capacity building approaches upstream from practitioner training. The majority of participants also noted a current and desired state of implementation support focused on trusting relationships, driven by community data, and centered in co-creative approaches where both intervention and implementation strategies are co-designed with community members.

**Figure 1.**
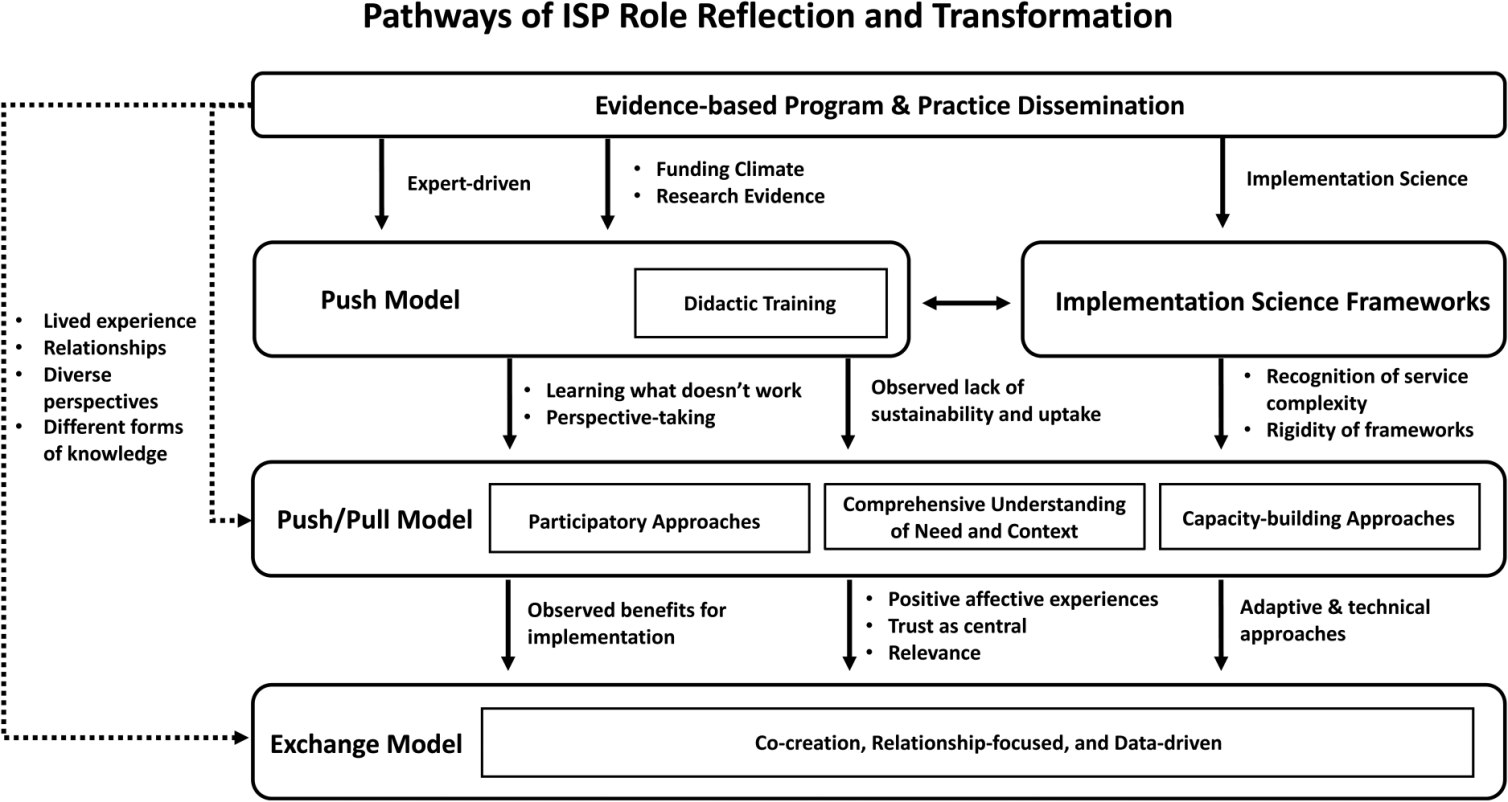
Visualization of pathways of implementation support practitioner role reflection and transformation.

Figure 1 depicts the pathways of ISP role reflection and transformation articulated by participants. Participants described their roots in providing implementation support^1^ in terms of the movement toward implementing and expanding the use of evidence-based programs and practices in child and family services in the late 1990s and early 2000s. The pathways ascending from this beginning are depicted in the figure through arrows. Pathways are unidirectional, representing a general linear progression in the changes to implementation support over the course of participants’ careers; with the exception of the introduction of implementation science theories, models, and frameworks that influenced first-order and subsequent approaches for evidence use. Dotted-line pathways represent a “leap frogging” across pathways, highlighting that not all participants started in the same place or moved through each pathway at the same pace or sequence. Contextual factors that enabled various pathways are featured next to each arrow, representing the influences and experiences that contributed to the participants’ narratives about changes in their implementation support practice.

### Push Model

Participants described the early influences of their use of a push model for supporting evidence use in child and family service systems. Participants explained a research and funding climate focused on disseminating programs and practices with evidence—one where experts were brought in to teach and train communities and organizations about the programs and practices that would improve outcomes for their local populations. They described limited stakeholder engagement and a rigidity in implementation support approaches. For all participants, the desire to improve outcomes reportedly drove their commitment to disseminating evidence-based programs through this expert driven approach in early stages of their careers as ISPs.

One participant reflected back on the top-down implementation support model so common 20 years ago, and the belief that evidence-based programs, regardless of context or acceptance and motivation of the local community, would improve outcomes.

In the earliest stages of this work 20 years ago, [we] said, ‘Hey, the goal is to scale up these evidence-based interventions because they’re evidence-based,’….it's like the tail wagging the dog. It's like they’re solutions looking for problems, and in fact sometimes they’re solutions that are being promoted irregardless [sic] of whether there is a problem that fits the solution.

The push model was described by participants as didactic and influenced by early implementation science strategies for supporting replication and scale-up. There was an emerging understanding that organizations and communities needed active support to uptake evidence-based programs and practices, and wide-spread URE in service settings would not be achieved through traditional dissemination activities. A participant described the rigidity of these early implementation support strategies as it related to supporting scaling of evidence-informed programs and practices.

…if we go back 15 years implementation support was usually centered around a very specific type of implementation framework…one organization had their very structured framework, and if they were engaging with a jurisdiction, the jurisdiction needed to be willing to fold itself into this framework and receive implementation support as prescribed.

As the field of implementation science evolved, there became a deeper recognition of the complexity of service systems and the need for flexible, multi-level implementation strategies. Participants described the shift from push models (i.e., training only) to more interactive forms of implementation support as the field of implementation science began to have more influence on dissemination strategies for evidence-based programs and practices.

…The field of implementation science was hardly a blip on the radar just yet. I spent a lot of time getting deployed out to train and help with different agencies elsewhere, and then seeing the very low levels of uptake that we had in these communities…It became apparent to me there was a substantial problem in the way that we were thinking about dissemination.

Figure 1 depicts the emergence of implementation science to support the dissemination of evidence-based program and practices and the evolution of implementation frameworks as a bridge between didactic training models to more sophisticated implementation support models that recognized the complexity of service systems and the need for flexible, multi-level implementation strategies.

### Push/Pull Model

Participants described moving away from training models to multi-level implementation support models that create space for peer learning and collaborative work. Although the fields of implementation science and evidence use have embraced multi-level approaches over the last decade, this is still a relatively new way of work for dissemination of evidence-based and evidence-informed programs and practices (e.g., Damschroder et al., 2015).

Participants emphasized shifting implementation practices related to engaging leadership, building teams, and assessing contextual fit of potential programs and practices. Participants also described an observed lack of sustained use of evidence-informed programs and practices using top-down, expert-driven models of implementation support. These observations, along with a deepening understanding of the importance of user-centered implementation support models, influenced shifts in the approaches of many participants from a push model to a push/pull model of implementation support. Participants described their experiences working intensively in communities and service systems as influential in their implementation practice, beyond what is included in implementation frameworks, theories, or strategies.

…Co-creation is not a well understood concept in the implementation world…It's [co-creation] an important shift in stance. We’re not the experts. We’re not here to tell you what to do. It takes experience to understand the art of knowing when the moment is right to do for and knowing when you need to back off and entrust people to do it themselves.

Participants with long-term experience providing implementation support (>10 years) described their own shift from training practitioners to engaging stakeholders at multiple levels of the system and developing opportunities for collaborative implementation planning and peer support. All participants perceived benefits in this shift. A participant described this shift from a push model to a more collaborative model that creates space for co-learning—involving both a push and pull of ideas among various implementation stakeholders.

…when I first started in the implementation support realm, I did a lot of stand-up training…I started off from a perspective of, “I have something to share,” and, “Here's something that would be useful for you sitting in the audience to hear.” And did not, in my early years, think about the reciprocal nature of implementation support and approaching it as a group of learners working together. That certainly shifted over time where I progressed to thinking about what it means to be a learner and what it means to be in a learning space together…I approach implementation support now more in line with the way I think about reflective supervision… facilitating learning as opposed to telling what the answer is…

Another participant shared a shift from a didactic training strategy to a community psychology model that involved conducting needs and resource assessments in service to helping the community make informed and strategic decisions about implementation of evidence-based programs and practices.

Participants noted the flaws in initial dissemination strategies that focused on didactic training methods not aligned with fundamental concepts of adult learning theory that require opportunities for peer-to-peer learning. Participants also noted that didactic implementation support did not empower end users and therefore limited the success of capacity building and sustainability efforts with service systems. A participant described moving away from an expert driven implementation support approach to an approach that recognized the needs and knowledge of end users.

My thinking about implementation support has shifted too…really trying to think more deeply about how to provide the right information at the right time in the right sort of way that meets the need of the people at that moment in time… Trying to think about what that end user experiences and what makes the most sense for people. So that has shifted over time…. that shift from thinking, “I’m the expert and you’re the learner,” to, “We are learning together.”

Another participant emphasized the shift from training practitioners in evidence-based programs (i.e., push model) to an implementation support model that involves multi-level support, a deeper understanding of context and enabling systems supports, and more focused implementation support upstream from practitioners.

Without pointing out where capacity needs to be built and helping them think through building that infrastructure so that the infrastructure remains, regardless of what intervention you’re trying to improve upon, you are just doing a point in time implementation of something that may or may not be sustained. It's a lost opportunity to prepare public systems to, overall, improve their operational functioning and regularly use evidence and data.

### Exchange Model

Participants observed benefits to implementation progress and sustainability as they shifted their approaches from expert-driven to collaborative. They described a growing emphasis on the relevance of intervention and implementation strategies for communities, rather than privileging evidence-based programs over community perceptions of need, capacity, or contextual fit. Participants also described a more explicit shift to relationship-building, noting a deepening understanding of the foundational role of trust in implementation efforts. Moreover, participants described their own affective experiences related to making these shifts, and the role of trusting relationships in their own implementation support practice.

…the idea of co-creation had been part of messaging for a number of years and I never really bit on that as being of primary importance…over the last eight to 10 years, I’ve really just become a dyed in the wool ambassador for the critical importance of co-creation.

The participant also described in detail an evolution in thinking that moved toward cultivating an internal drive to engage in implementation work among those being supported:

The most successful examples I’ve had in this work were because I, rather than going in as the expert in some subject area and imparting knowledge on people, I instead went in and asked probing questions to encourage them to think about what their issues are, what might be the strategies to resolve those issues…I’ve found it more valuable…to foster their own curiosity and their own commitment to wanting to do better.

Participants shared how they intentionally moved away from a top-down approach to co-creating with the people in the service systems who will actually need to do the work. All participants perceived this shift as beneficial for implementation and evidence use, noting that co-creation cultivates an internal drive for those with the greatest stake in implementation efforts to engage in implementation and improvement efforts. For example, one participant described centering the desires of local organizations in the selection of measures and collection and interpretation of data and how this has led to successful co-design of community-based approaches that are culturally relevant. Another participant described how implementation support should not involve leading people to specific models or approaches, rather it should involve providing just enough information to help local stakeholders make their own decisions. Participants described their role as “meaningfully engaging people in the field” and supporting them to understand and define the problem they want to address and the potential solutions to be considered and ultimately selected.

Participants shared how they have pivoted away from the use of specific frameworks or methods, focusing more on developing trusting relationships and building teams. For example, participants described using specific implementation science frameworks or improvement methods (e.g., breakthrough series), and while these tools provided some benefit, participants shared that building trust within teams was one of the most important factors for implementation success.

The one thing that I do know, or that I wholeheartedly believe is a definite, is none of the implementation strategies will work without relationships in jurisdictions, right? You can't go into a jurisdiction and just sort of fly in and say, “We’re gonna do these things,” without having built trust, built a relationship and really being seen as a trusted partner, that's sort of widespread.

Participants described becoming more intentional in their development of leadership teams and teams with broader stakeholders and community members. Participants also described how important it was that ISPs are supported by a team of peers that they trust and with whom they can troubleshoot implementation support challenges. One participant shared, “teams are, in the end, what makes a project go, and so we have to create teams.”

Another participant shared how data can be used to support collaborative decision-making among teams and also ensures that ISPs are humble, curious, and transparent when they are facilitating teams.

I’m just a believer that data is the great convener…the more we can work as the university partner to help them understand how to read data, the more we sit there transparently and hear why they think….there's an implementation reason that we’re not catching in the data and really be humble and transparent when they push back on it. I feel like it's just this ever evolving willingness to listen and be heard, expect people to hear us, and hear the hard stories data tell…why do the data look like that?

Some participants described starting in a place further along the pathway depicted in Figure 1, relative to the majority of the participants interviewed. Participants reflected on their “starting point” in providing implementation support, and those who started using a push/pull or exchange/co-creation model earlier in their careers described coming into their roles as ISPs with the experience of having served as program leaders or staff and received technical assistance or implementation support that was not helpful. Early experiences receiving suboptimal or ineffective implementation support propelled a subgroup of ISPs to leap over the starting point of a push model described by the majority of ISPs and to immediately embrace more responsive, contextualized, and relationship-based strategies.

## Discussion

Findings from this study suggest that ISPs have evolved their role and approach based on their professional experiences, emerging knowledge about implementation and scaling practices, and their observed benefits and challenges of using specific implementation models and strategies. The three main implementation support approaches were push, push/pull, and co-creation/exchange. ISPs interviewed for this study described these pathways and made their own connections regarding how their approaches to implementation support have changed over time and why they have made these changes. It is possible that additional pathways and connections exist, depending on a range of variables. The study sample included relatively experienced ISPs in terms of providing implementation support for evidence use in child and family service contexts. As the field of implementation science evolves and the workforce grows, contextual factors such as professional training and education of ISPs will likely impact starting points and pathway connections for professionals in this role.

The role of ISPs in building implementation capacity in service agencies has gained increasing attention, with recent publications describing the knowledge, skills, and attitudes needed to be effective in this capacity building role (Albers et al., 2021; Metz et al., 2021); the strategies for building a competent workforce through the design of professional training and coursework (Moore et al., 2018; Mosson et al., 2019; Park et al., 2018); and the evidence for specific approaches to implementation capacity building (Leeman et al., 2017; Stander et al., 2018). However, gaps remain in the implementation science literature regarding the experiences of ISPs and how their interactions providing implementation support contribute to the evolution of implementation capacity building approaches for evidence use and moving beyond the historical and ongoing emphasis on tools and checklists.

Taxonomies of implementation strategies cannot fully account for the complex process of implementation, which involves a range of different actors with different capacities and skills across multiple system levels. Taxonomies such as the compilation produced by the Expert Recommendations for Implementing Change project (Powell, et al., 2012; Powell et al., 2015) are valuable implementation tools as they support the selection, use, and improvement of implementation strategies to achieve implementation outcomes in a range of contexts. However, the basic elements described for supporting implementation remain broad. As observed by Albers et al. (2021) based on their systematic review of implementation strategies, there is ample room for further operationalizing and tailoring of the strategies.

The experiences of ISPs, as described in the current paper, provide one possible source for further defining the activities and functions for providing implementation support. For example, study participants identified developing trusting relationships as an important strategy that emerged through their experience providing implementation support, which aligns with other recent research findings (Akin, 2016; Albers et al., 2021; Barac et al., 2018; Bührmann, et al., 2022; Metz et al., under review). Insights from the experiences of ISPs can enrich implementation research to demonstrate the range of approaches for building implementation capacity and the rationales for evolving approaches that emphasize the dynamic and highly relational nature of using evidence in practice and the multiple layers of context and range of stakeholder groups involved in the process (Albers et al., 2021; Carey et al., 2019; Huzzard, 2021).

The sample of ISPs interviewed for this study demonstrated the use of implementation strategies that require a range of skills including technical skills, such as using data and conducting improvement cycles, as well as relational skills including building trust between themselves and among stakeholders and brokering relationships across siloed stakeholders. Study participants described an evolution to their implementation capacity building approaches, and the specific strategies they used over time, representing a multi-faceted, emergent approach to implementation support, centered in reflective practice, ongoing learning, and cumulative knowledge development from previous successes and failures to achieve implementation outcomes.

Indeed, study participants were able to describe changes in their own implementation support approaches over time and provide rationales for these shifts grounded in observations related to whether underlying assumptions for effective implementation support “held up” in real world settings. For example, participants observed flaws in the logic that evidence-based practices could be scaled through widespread trainings, leading to the use of participatory approaches that embraced complex systems, variations in contextual fit, and the need for multi-level capacity building to achieve implementation outcomes. As participants recognized benefits to participatory approaches, they tested new approaches focused on co-creation and relationship-building, with the intention of increasing the relevance and sustainability of implementation capacity building approaches in a range of settings. These findings indicate that implementation research may be out of step with implementation practice.

In order to understand *how* ISPs can support the implementation of change in practice (Albers et al., 2020), it is important to systematically collect observations of and insights from professionals doing this work. In doing so, the field of implementation science will better understand the mechanisms for change (Lewis et al., 2018) in service settings, namely the connections between implementation capacity building approaches and implementation outcomes. Systematically studying the experiences of professionals supporting implementation efforts is likely to contribute to a growing understanding of the resources and strategies needed to build sustainable implementation capacity and evoke positive affective responses from stakeholder groups involved in implementation efforts.

As the field of implementation science seeks to identify the skills, knowledge, and attitudes of effective ISPs (Bührmann, et al., 2022), deepening our understanding of the factors and conditions ISPs perceive as critical to implementation success will help to build a workforce of professionals positioned to deliver effective implementation support.

## Conclusions

The analysis presented in this paper suggests that implementation science stands to benefit from a stronger connection between implementation research and implementation practice. Whereas implementation research seeks to understand the approaches that work best to translate research to the real world, implementation practice seeks to apply and adapt these approaches in different contexts to achieve outcomes (Ramaswamy et al., 2019). The creation of a virtuous learning cycle between professionals conducting implementation research and professionals providing implementation support to change practice is likely to produce a more robust and relevant science of implementation. This paper foregrounds the critical question of whether the lived experience of ISPs can enable that virtuous cycle by providing detailed information on “what it takes” to effectively build implementation capacity, engage with stakeholders, and achieve sustainable implementation outcomes in a range of settings, so that all people and communities can benefit. Developing a workforce that provides implementation support will require a deeper understanding of this lived experience to prevent repeated use of strategies observed to be unsuccessful by those most proximal to the work. The emergence of pathways for implementation practice in this study highlights the impressive leaps forward the field of implementation has taken in the last 15 years and speaks to the critical role of professionals leading change efforts in this growth.

## Notes

1. Participants used the terms implementation support and technical assistance interchangeably. To increase clarity in presenting results, the term implementation support is used consistently.
